# How to Leave Church

**Published:** 1856-05

**Authors:** 


### HOW TO LEAVE CHURCH.

Some one has said, that every fierce burst of passion, every exhibition of unbridled rage, shortens human life. We all know that men have died in a fit of passion; and what destroys life in its intensity, must shorten it in its modified exhibitions. The shock of an unexpected item of news, has many a time sent the startled recipient to the judgment. And there is hidden meaning in the Divine inquiry, *A wounded spirit who can bear?* How many an eye, bright and beautiful as the sun light, has paled away into the grave, from blighted affection. These known facts compel us to admit what a large influence the states of the mind have on bodily health, and press home on the heart and conscience of the noble few who make the *law of love to one another* the guiding star of life, the duty, the humanity, the blessing, of so shaping their words and actions, at home and abroad, in the private circle and in the public assembly, as to be least liable to "*offend*," to chafe, to wound, to shock, those who are about them. Were we all to do so, how many a sad heart would be less sad, how often might we plant a flower, where else we find a thorn! To make this applicable to the heading of this article, we have only to record our own experience of the ugly, grating harshness which swept across us in those other years when we were younger and better than now, at the criticism some one made on a sermon, in passing out of the church. It was the sermon of a giant in grace and intellect; of one who was just stepping into the grave, he seemed to be speaking back to us from the other side of the tomb, and while his tones of tenderness and grandeur were still thrilling our heart, through the prayer and hymn, and solemn benediction, the expression fell upon the ear like Milton's doors on rusty hinges turning,

"Grating harsh thunder."

"It was a pretty good thing that sermon, wasn't it?"

As we cannot say that some tender chord has not been touched in any given sermon or address, and as it is quite certain that in every religious exercise some heart has been more or less dissolved to tenderness and some eyes to tears, we suggest, the policy, the wisdom, the beneficence of a rule: *In passing from a place of religious worship, do nothing by word or gesture or action, incompatible with the solemnity of God's peculiar presence.*

